# Nevus of Ota with Rare Palatal Involvement: A Case Report with Emphasis on Differential Diagnosis

**DOI:** 10.1155/2011/670679

**Published:** 2011-07-28

**Authors:** Gaurav Sharma, Archna Nagpal

**Affiliations:** ^1^Department of Oral Medicine and Radiology, Sudha Rustagi Dental College, Faridabad 121002, India; ^2^Department of Oral Medicine and Radiology, PDM Dental College, Bahadurgarh 124507, India

## Abstract

Nevus of Ota, a dermal melanocytic nevus, is rare in the Indian subcontinent. It presents as a brown, blue, or gray patch on the face and is within the distribution of the ophthalmic and maxillary branches of the trigeminal nerve. The oral cavity is infrequently involved in nevus of Ota. Only 11 cases have been documented in the English literature. We report a rare case of intraoral nevus of Ota in a 22-year-old male patient. This paper focuses on the differential diagnosis of oral manifestations of nevus of Ota to assist in proper followup to avert malignant transformation.

## 1. Introduction

The nevus of Ota or oculodermal melanocytosis is a macular discoloration of the face which is most commonly found in the Japanese [1]. Nevus of Ota, first described by Ota and Tanino in 1939, involves the skin along the distribution of first and second division of trigeminal nerve [2]. It is a rare condition that affects only 0.014%–0.034% of the Asian population and is rare among males [3]. The onset of nevi typically is either at birth (60%) or soon after birth, but few rare cases of acquired type have also been reported. The involvement of oral mucosal membrane is extremely rare. We report a rare case of nevus of Ota in a 22-year-old male with palatal involvement in an Indian population.

## 2. Case Report

**Figure 1:**
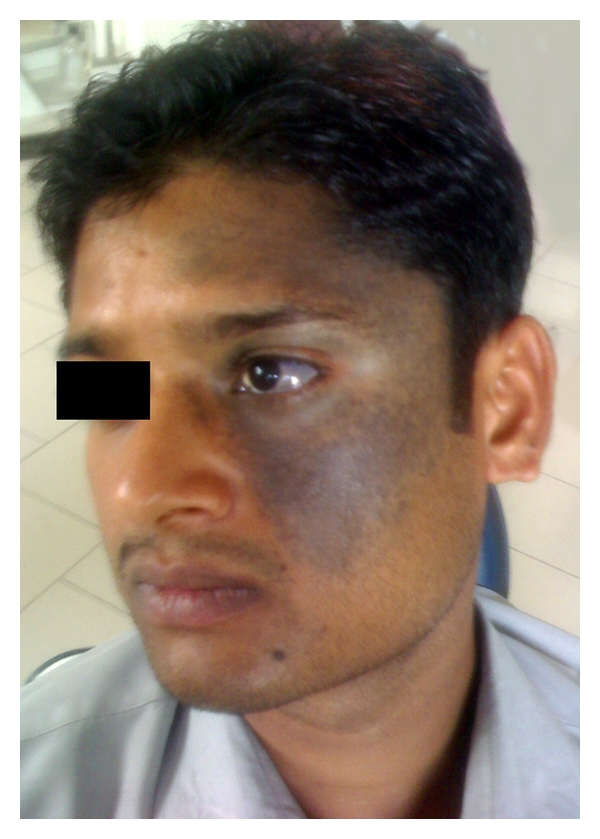
Extraoral involvement of nevus of Ota.

A 22-year-old male presented to dental OPD with the chief complaint of stains on teeth. There was no significant medical history reported. He had asymptomatic, bluish, speckled, coalescing and well-demarcated hyperpigmented macules on the left midface extraorally (Figure 1). It was involving temporal, frontal zygoma and the maxillary sinus area. Bluish pigmentation of sclera was also observed. On eliciting specific history regarding scleral and facial discoloration, the patient revealed that it was present since birth, and family history was noncontributory. The discoloration gradually had increased till the age of 14 years and was static ever since. Patient denied taking any medication, and he gave no history of photosensitivity. Patient was asymptomatic with no effect on vision, sensory changes, and hearing. 

**Figure 2:**
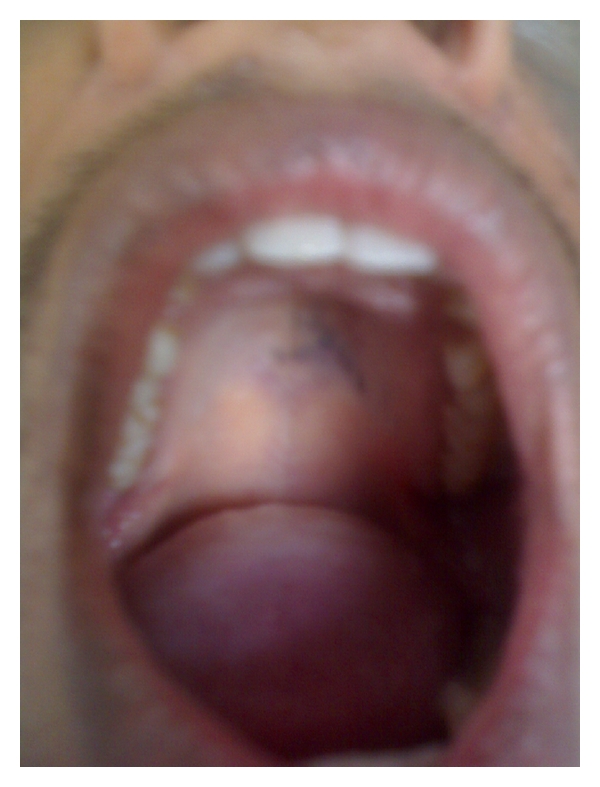
Intraoral involvement of nevus of Ota involving hard palate.

On intraoral examination, bluish discoloration approximately 2 cm in diameter with diffuse margins was evident on the left side of hard palate, which was somewhat crossing the midline (Figure 2). The buccal mucosa, tongue, and floor of mouth were normal. Patient was referred to dermatology for consultation, which did not report any abnormality other than discoloration of face. Auditory examination also did not reveal any discoloration of the auricle and tympanic membrane. No abnormality other than discoloration was detected following ophthalmologic examination. A clinical diagnosis of nevus of Ota was given with the consensus of dermatologist. The patient was advised treatment for the same, but he was not willing for any kind of treatment as he was asymptomatic. Patient was subsequently referred for oral prophylaxis and was advised to report for regular followups of nevus of Ota.

## 3. Discussion

The exact etiology of nevus of Ota is still unknown. However, nevus of Ota may represent melanocytes that have persisted and not migrated completely from the neural crest to the epidermis during the embryonic stage. Other theories which have been postulated is the active production by intradermal melanocytes [2]. 

Clinically, nevus of Ota presents as a brown, blue, or gray patch on the face, which is congenital or acquired and is within the distribution of the ophthalmic and maxillary branches of the trigeminal nerve. Women (80%) are affected more frequently than men, and the nevus can be unilateral or bilateral, but unilateral involvement is common (90%–95%) [4]. Nevus of Ota is asymptomatic though rare cases of sensory loss have been reported [5]. Extracutaneous sites like eyelids, and their adjacent skin areas, sclera, and conjunctiva, have also been involved. Spontaneous regression does not occur, although the intensity of the pigmentation may vary in relation to menstruation, fatigue, or weather [6]. 

Nevus of Ota often occurs in association with nevus of Ito, which is a dermal melanocytic condition affecting the shoulder area [7]. Nevus of Ota can also be associated with other cutaneous disorders and ocular disease. Benign cutaneous and leptomeningeal conditions associated with nevus of Ota are phakomatosis pigmentovasculari, nevus flammeus, Sturge-Weber syndrome, Takayasu disease, Klippel-Trenaunay syndrome, and neurofibromatosis [5]. 

Mishima classified nevus of Ota into three types, depending on the extent and distribution of pigmentation [6]. Thus, our case will correspond to Type III classification, as there was involvement of areas innervated by second and third divisions of trigeminal nerve (Table 1).

Clinical differential diagnosis for skin lesions of nevus of Ota includes mongolian spot, melasma, blue nevus, and drug-induced hyperpigmentation. In mongolian spot, there are poorly demarcated large blue-to-grey patches that tend to spontaneously resolve by age 3–6 years and typically tends to occur in lumbosacral area and rarely in face. Melasma is typically associated with pregnancy and clinically appears as well-to-poorly demarcated and irregularly outlined brown-to-gray brown patches. Melasma typically is bilateral and there is no palatal involvement. 

Drug induced hyperpigmentation is usually acquired after ingestion of drugs like minocycline, amiodarone and gold. Blue nevus typically appears as plaque or papules which may occur anywhere on skin and can be differentiated from nevus of Ota which has typically macular presentation. Sclera and oral mucosa are not involved in acquired bilateral nevus of Ota like macules (ABNOM) or Sun's nevus. 

Oral melanotic macule can also be misdiagnosed as nevus of Ota. It can also exist in palate but can be distinguished from nevus of Ota as it is commonly smaller in size, and there is no involvement of sclera. Oral melanotic macule can occur solitary or as a part of syndromes like Peutz-Jeghers syndrome, LEOPARD syndrome, and Laugier-hanziker syndrome. Our patient, however, had no pigmentation of nail, perioral pigmentation, lentigines, and deafness, thus exclusion of above syndromes was made. 

There is no definitive diagnosis for nevus of Ota. Diagnosis is mainly by clinical examination and history. Skin biopsies are required only if clinical changes are suspected of malignant transformation (e.g., ulceration, new papular lesions, and variegations in color) within the involved skin, ocular tissues, or mucosal tissues.

Involvement of palatal mucosa occurs infrequently in nevus of Ota. So far, only 11 cases of nevus of Ota with intraoral involvement have been reported (Table 2) [2]. Most of cases have occurred in females (64%). Appearance of palatal pigmentation usually blends with oral mucosa and is typically irregular, ill defined, and often mottled patch [2]. Palatal pigmentation in nevus of Ota can be differentiated from blue nevus as blue nevus is slightly raised or papular lesion that is usually less than 1 cm in size. Biopsy is generally not indicated.

Malignant melanoma has also been reported in patients with nevus of Ota [8]. Most of cases of malignant melanoma developing from nevus of Ota are mainly found in skin. Less commonly, involvement of meninges and ocular area has also been reported. However, no cases of malignant melanoma in oral cavity developing from nevus of Ota have not been reported. Nevertheless, with the cases of malignant melanoma being reported in other areas, the risk of malignant melanoma in oral cavity cannot be ignored because of the scarcity of reported cases. All the patients of nevus of Ota should be referred to dentists for the risk of oral mucosal involvement, and they should be screened biannually for any signs of malignant transformation like sudden increase in size of discoloration, ulceration, and paresthesia.

The sclera is commonly involved in patients with nevus of Ota. The ocular complications associated with nevus of Ota are increased intraocular pressure and glaucoma [9]. Melanoma arising in orbit, iris, ciliary body, and optic nerve in association with nevus of Ota has been documented [8]. Periodic reevaluation with ophthalmologist should also be performed annually.

 Topical therapy is of no value in the medical treatment of nevi of Ota. Previous treatment modalities, including cryotherapy, dermabrasion, and microsurgery, can be associated with scarring [3]. Development of the Q-Switched Nd: YAG laser (QSYL) and the Q-switched ruby laser (QSRL) has enabled complete, scarless elimination of the pigmentation in patients with nevus of Ota. Without treatment, the skin lesions are permanent [10].

From a dental practitioner's point of view, the condition that the dental practitioner can also easily confuse with nevus of Ota because of lack of familiarity about this condition is hemangioma. Hemangioma will typically not involve sclera as seen in nevus of Ota. Another similar condition involving the same extent of area that can be confused with nevus of ota is port wine stain (which maybe a part of Sturge-Weber syndrome), which can be differentiated from the color of appearance. Therefore, the dentist should have awareness about nevus of Ota so that there are no unnecessary investigations like ultrasonography or angiography for the patient if he is suspected for hemangioma. 

Dentists should inform the patient about the condition and should be trained to detect any atypical change in size, color, and morphology. A better association between dentists and dermatologist is required as patients will primarily visit a dermatologist initially for the facial disfigurement. It, thus, becomes imperative for a dentist to know the differential diagnosis of the oral manifestations of nevus of Ota as patient will be referred to dentist for intraoral examination.

Pigmented lesions are commonly seen by dentists. The purpose of writing this paper is to demonstrate the variation in the presentation of pigmented macule. The lack of awareness of nevus of Ota can lead to misdiagnosis and, hence, an unknown risk for malignant transformation as there will be no regular followup.

## 4. Conclusion

Nevus of Ota with palatal involvement in males is a rare entity. Dentists should be ever alert to diagnose this condition and refer the patient for dermatological and opthalmological consultation, as malignancy has been documented. Patients with nevus of Ota and especially with palatal pigmentation should always be examined by dentists. Dentists should have a thorough knowledge of nevus of Ota and its differential diagnosis to avoid misdiagnosis and, hence, the risk of malignant transformation, thereby avoiding any type of medicolegal concerns. Patients should be taught awareness of the potential of glaucoma and malignancy. A multidisciplinary screening involving dermatologist, ophthalmologist, and dentist is suggested for patients afflicted with nevus of Ota.

## Figures and Tables

**Table 1:** Classification of nevus of Ota (Mishima's classification).

Subtypes	Intensity	Pigmentation	Area involved
Type I	Mild	Light brown	Upper and lower eyelids and zygomatic area
Type II	Moderate	Deep slate Gray	Eyelids, zygomatic area, and base of nose
Type III	Intensive	Deep blue to brown	Affecting the first and second divison of trigeminal neuralgia

**Table 2:** Chronological listing of intraoral nevus of Ota (Documented cases).

Case	Author	Gender/age	Location
1	Dorsey and Montgomery	M/16	Buccal mucosa
2	Mishima and Mevorah	F/35	Hard palate
3	Mishima and Mevorah	M/45	Hard palate
4	Decosta and Carneiro	M/23	Buccal mucosa
5	Reed and Sugarman	F/43	Hard palate
6	Yeschua	F/27	Buccal mucosa
7	Page	F/59	Hard palate
8	Rathi	F/30	Hard palate
9	Karthiga	F/32	Hard palate
10, 11	Parihar	F/32, M/33	Hard palate
12	Present author	M/22	Hard palate
